# The Role of Dyslipidemia in Colitis-Associated Colorectal Cancer

**DOI:** 10.1155/2021/6640384

**Published:** 2021-02-12

**Authors:** Ke Chen, Jianrong Guo, Tao Zhang, Jian Gu, Huili Li, Jiliang Wang

**Affiliations:** ^1^Department of Gastrointestinal Surgery, Union Hospital, Tongji Medical College, Huazhong University of Science and Technology, Wuhan 430022, China; ^2^Department of Gastric Surgery, Sun Yat-sen University Cancer Center, Guangzhou, China; ^3^Department of Anesthesiology, Union Hospital, Tongji Medical College, Huazhong University of Science and Technology, Wuhan 430022, China

## Abstract

Dyslipidemia, characterized by metabolic abnormalities, has become an important participant in colorectal cancer (CRC). Dyslipidemia aggravates intestinal inflammation, destroys the protective mucous layer, and disrupts the balance between injury and recovery. On the other hand, antioxidants induced by oxidative stress enhance glycolysis to maintain the acquisition of ATP allowing epithelial cells with damaged genomes to survive. In the repetitive phase of colitis, survival factors enable these epithelial cells to continuously proliferate. The main purpose is to restore and rebuild damaged mucosa, mainly aiming to recover mucosal damage and reconstruct mucosa, but it is also implicated in the occurrence and malignancy of CRC. The metabolic reprogramming of aerobic glycolysis and lipid synthesis enables these transformed epithelial cells to convert raw carbohydrate and amino acid substrates, thereby synthesizing protein and phospholipid biomass. Stearoyl-CoA desaturase, responsible for the fatty acid desaturation, improves the fluidity and permeability of cell membranes, which is one of the key factors affecting metabolic rate. In response to available fat, tumor cells reprogram their metabolism to better plunder energy-rich lipids and rapidly scavenge these lipids through continuous proliferation. However, lipid metabolic disorders inhibit the function of immune-infiltrating cells in the tumor microenvironment through the cross-talk between tumor cells and immunosuppressive stromal cells, thereby providing opportunities for tumor progress. Nonsteroidal anti-inflammatory drugs and lipid-lowering drugs can decrease the formation of aberrant crypt foci, lower the burden of the adenomatous polyp, and reduce the incidence of CRC. This review provides a comprehensive understanding of dyslipidemia on tumorigenesis and tumor progression and a development prospect of lipid disorders on tumor immunity.

## 1. Introduction

Colorectal cancer (CRC) remains the third most common type of cancer and the second leading cause of cancer-related death worldwide [1], especially among people who engage in a western pattern diet without appropriate physical exercise [2, 3]. In recent years, the mortality and incidence of CRC have been consistently increasing, which is related to many factors. Dyslipidemia is a condition of abnormal lipids, such as high triacylglycerol, cholesterol, and low-density lipoprotein cholesterol levels, which is characterized by metabolic disorders highly associated with obesity [4]. It is often due to diet and occurs under several pathological conditions, such as atherosclerosis, nonalcoholic fatty liver disease, or long-termed administration of drugs such as rosiglitazone to treat type 2 diabetes [5]. We have mentioned that, clinically, the resected neoplasm of CRC is usually infiltrated by white adipose tissue, regardless of body size. At the same time, large numbers of epidemiological studies have shown that obese people with dyslipidemia, especially those with hyperlipidemia, have a high risk of prostate cancer, lung and bronchus cancer, CRC, melanoma, and ovarian cancer. Previously, many studies have mainly focused on the impacts of dyslipidemia on tumor progress [6] or specific lipid component change of CRC tissue. In addition, the existing research is mainly concentrated on conventional adenomas-derived carcinoma and only deals with one aspect of dyslipidemia on cancer, which is not comprehensive. Nevertheless, colon cancer may also originate from inflammatory bowel disease. Dyslipidemia is associated with long-termed intestinal inflammation [7].

Dyslipidemia will strengthen the inflammatory response and have many effects on the occurrence and development of colitis-associated CRC in patients with inflammatory bowel disease. Chronic intestinal injury destroys the protective mucus layer and mucosal epithelium, thus disrupting the balance between intestinal injury and recovery [8]. Accumulated genetic alteration evolves cancerous transformation. In addition, in the progress of colorectal cancer, which is significantly different from that of normal mucosal epithelium, cell proliferation and survival require reprogramming metabolism of lipogenesis-related enzymes [9]. Tumor lipid metabolism reprogramming affects the systematic lipid level as well. Although the prevalence of dyslipidemia is positively correlated with the incidence of colitis-associated CRC, the potential relationship is not completely clear. Previous studies were zooming in on a certain field of abnormalities of lipid disorder on tumor growth, invasion, and distant metastasis and were not focused on the occurrence of colitis-associated CRC and its progress [10–12]. In view of the fact that dyslipidemia is an important part of obesity and the high prevalence of diseases related to dyslipidemia worldwide, the causal association between dyslipidemia and colitis-associated CRC should be clarified. This paper summarizes and evaluates the existing evidence about dyslipidemia and colitis-associated colorectal tumorigenesis and progress, discusses the influence of dyslipidemia-related conditions on colitis and the resulting tumorigenesis, and analyzes the effects of tumor metabolic reprogramming and the tug-of-war between tumor and infiltrating immune cells in response to high fat, to have a more comprehensive understanding and to provide early intervention measures.

## 2. Dyslipidemia Accelerates Colitis-Associated CRC

### 2.1. High-Fat Uptake of Intestinal Epithelial Injury

Infectious agents, chemical stimulus irritating chronic colitis, or even some autoimmune diseases destroy the balance between intestinal mucosal injury and recovery. Though the gut is teeming with numerous bacteria, gastroenterology has a solid line of defense against pathogenic microbes [13]. Bowel inflammation is related to mucosal epithelial injury. When confronted with persistent irritants, activated macrophages move around and swallow agents and then release IL-17 to recruit polymorphonuclear cells and other immune cells to participate in agent elimination [14], ultimately resulting in a microenvironment composed of necrosis tissues, immune cells, stromal cells, and cytokines. High-fat diets contribute to enhanced bowel inflammation partly due to the strengthened metabolic endotoxemia. Saturated lipids play an important role in accelerating inflammation through direct lipotoxicity, activation, or transformation of toll-like receptor-4 into bioactive forms [15]. A large intake of fat altered mucosal permeability and increased the absorption of lipopolysaccharide. Continuous serum lipopolysaccharide in the diet causes postprandial endotoxemia and inflammatory pathway activation, which in turn accelerates mucosal epithelia damage and forms a vicious circle. Crohn's disease was positively correlated with the prevalence of nonalcoholic fatty liver disease [16]. Obese people with fatty liver disease seem to be more susceptible mainly due to the lower detoxification of steatotic hepatocytes. In addition, long-lasting lipopolysaccharide stimulation can cause lipid changes and aggregates aberrant lipid metabolism [17].

Lipid abnormality has been long involved in enhanced inflammatory responses, which focus on the peroxidation of lipid and the increase of reactive oxygen species (ROS). These hyperactive oxidants are mainly produced by respiratory burst of polymorphonuclear cells. The imbalance of scavenging capacity leads to the oxidative stress of colonic mucosal epithelial cells in the microenvironment and induces DNA damage [18]. However, long-term hypoxia microenvironment can also induce increase in nuclear factor (erythroid-derived 2)-like 2 and hypoxia-inducible factor 1*ɑ* [19], which decouples fatty acid *β*-oxidation with the tricarboxylic acid cycle, inhibiting the production of ROS, and promotes glycolysis to maintain ATP, thus helping the damaged mucosal epithelium survive.

### 2.2. Dyslipidemia and Occurrence of Colitis-Related Tumors

A previous study showed that high-fat consumption was accompanied by an increase in the expression of *β*-catenin and the emergence of carcinogenic bacteria *Lachnospiraceae/Streptococcaceae* in the large intestine of mice [20]. A high-fat diet can aggravate the inflammatory effect of 1,2-dimethylhydrazine [21] and the carcinogenic effect of dextran sulfate sodium plus azoxymethane [22, 23]. In contrast, inflammatory bowel disease (IBD) is closely related to the occurrence of colorectal tumors and is one of the common precancerous lesions. A diet rich in animal fat and low in fiber increases the risk of IBD. Dietary fat can be a source of other types of lipids. Western diets are also linked to higher bile acids in the intestinal cavity, which is due to damage to the bile acid transporter in the epithelium [24]. In addition, the transformation of cholesterol metabolites of bile acid by intestinal bacteria can directly promote the proliferation of colonic epithelial cells or change the gene structure, induce colonic crypt abnormality [25, 26], and may aggravate the pathogenesis of colitis-associated CRC. Moreover, deoxycholic acid accumulation induced by high-fat intake increased the number of adenomas in Apc^Min/+^ mice [27]. In contrast to omega-3 fatty acids, abnormal dietary omega-6 fatty acids accelerate the progress of Crohn's disease, mainly due to their conversion to arachidonic acids. A combination of abnormal omega-6 fatty acids with the aberrant profile of unsaturated lipid metabolism enzymes increases the susceptibility to Crohn's disease in children [28].

The location of lipid deposition may also influence the progress of colitis. Visceral fat depot, especially mesenteric fat, significantly promotes the translocation of gut bacteria of Crohn's disease [29], resulting in the accumulation of inflammation. Interestingly, long-lasting intestinal inflammation is also highly associated with CRC, especially in patients with visceral obesity. Adipocyte malfunction plays an important role in facilitating systemic metabolic disorders and inflammation. The pathogenesis of metabolic syndrome may be related to the accumulation of visceral adipose tissue, which is characterized by the convergence of macrophages, immune cells, and stromal cells, leading to adipocyte malfunction and abnormal release of adipokine [3]. The infiltrated M2 subtype macrophages in the microenvironment exhibit a phenotypic switch from anti-inflammatory to pro-inflammatory properties and secrete inflammatory cytokines, resulting in systemic chronic low-grade inflammation [30]. In addition to adipokine disorder, chronic endotoxin can also cause or aggravate systemic metabolic disorders. In addition, sphingosine kinase 1 and sphingosine-1-phosphate, a downstream product of the sphingomyelin metabolic pathway, play a role in NF-*κ*B-dependent inflammatory activation and accelerate colitis-associated colorectal tumors [31].

The inflammatory microenvironment is mutagenic. Hyperlipidemia participates in the strengthened inflammation response by excessive lipid peroxidation or epigenetic modification, which accelerates the accumulation of oncogene mutations and promotes the occurrence of inflammation-associated colorectal tumors. The lack of adequate DNA repair of damaged colonocytes can lead to tumorigenesis. Abnormal lipids change the body homeostasis and amplify the ROS/RNS spectrum, thus damaging DNA, to accelerate the activation of proto-oncogenes (such as Ras) and the inactivation of suppressor genes (such as P53), resulting in the loss of cell cycle control and initiation of the tumorigenesis [32]. At the same time, the accumulated gene mutations of the damaged epithelial gain continuous abnormal proliferation capacity and exhibit a trend towards heterogeneity, especially for epithelial cells susceptible to neoplasia and precancerous lesions such as ulcerative colitis (UC) and familial adenomatous polyposis (FAP). Previous research by Chakrabarty et al. [33] has identified several gene mutations in CRC associated with ulcerative colitis, of which TP53 (17%), KRAS (22%), APC (33%), and RAF1 (39%) have been observed. Another relevant research by Setia et al. [34] showed that the activation of MAPK and PI3K pathways was also involved. Certainly, colonic Crohn's disease is different from UC in that it involves unique hub gene expression. In addition, aberrant proinflammatory interleukin-6 is a powerful tumor promoter in the early stage of solid tumor formation [35]. Interleukin-6 can enable the mutagenic epithelial cells to survive and accelerate cellular proliferation in colitis-related tumorigenesis. Crohn's disease is characterized by remitting and relapsing depending on the progress of inflammation. During the interval of the acute episode, both the chronic inflammation response and prosurvival factors provide the epithelia with the capacity of constant proliferation, which mainly aims to recover mucosal damage and reconstruct mucosa [36]. Nevertheless, high fat can activate the intestinal mucosal stem and progenitor cells and augment the self-renewal capacity via a peroxisome proliferator-activated receptor delta dependent manner [37]. Uncontrolled cell proliferation indicates the initiation of tumorigenesis.

### 2.3. Obesity-Associated Insulin Resistance

Excess lipid deposition within the skeletal muscle is the main cause of insulin resistance. High levels of fatty acids or lipid metabolites, especially oxidized low-density lipoprotein, can activate CD36^+^ cells and at the same time reversely inhibit insulin-associated glucose transporter-4, thereby inducing insulin resistance, which is defined as obesity-associated insulin resistance [38, 39]. Stimulation of high fatty acid load will accelerate the secretion of insulin in pancreatic *β*-cells, but, due to the impaired insulin sensitivity mechanism, the physiological functions of insulin are almost ineffective. Hyperinsulinemia is involved in tumorigenesis and malignant transformation. Insulin and insulin-like growth factor (IGF) have similar homologous fragments and other synergistic effects. Both are important determinants of cell proliferation and can mediate regulated cell death [40]. Dynamic increase of insulin and IGF has been observed in the process of carcinogenesis from polyps to adenomas and adenocarcinomas [41]. The activation of the insulin and IGF system exerts many functional effects on the colonic mucosal epithelium, leading to increased proliferation and survival of the Ras/MAPK pathway and significantly provoking tumorigenesis by the Akt/mTOR pathway [39].

In addition, obesity-associated insulin resistance can aggravate glucose and lipid metabolic disorders. Abnormalities of sugar and lipid are often the key to tumor metabolism. They may form a vicious circle, which will eventually promote tumor progress. Hyperglycemia is positively correlated with electron leakage in the mitochondrial electron transport chain, which is closely related to DNA damage and the resultant tumorigenesis [42]. Individuals with higher fasting glucose, insulin, triglycerides, and total cholesterol are closely associated with the risk of colorectal neoplasm. Synergy effects by glucose intolerance and abnormal lipid metabolism accelerate the occurrence and development of colorectal neoplasm [43, 44]. In addition, visceral adipocytes and body mass index play a role in the development of a colorectal neoplasm. The study of Del Cornò et al. [45] showed that visceral adipocytes of obese or CRC individuals were involved in pathways related to the metabolism of pyruvate, lipids, and glucose and processes relevant to carcinogenesis. It indicated that visceral adipocytes may participate in the establishment of a favorable tumor microenvironment.

### 2.4. Anti-Inflammatory Drugs

The administration of nonsteroidal anti-inflammatory drugs (NSAIDs) lowers the incidence and remits the malignance of CRC, which provides conclusive evidence for the close relationship between chronic intestinal inflammation and tumorigenesis. Eicosanoid metabolites from arachidonic acid under the action of cyclooxygenase or lipoxygenase participate in cellular signaling transduction as lipid mediators, resulting in both pro- and anti-inflammatory effects, and are associated with the occurrence of colitis-associated tumorigenesis [46]. The increase of cyclooxygenases-2 in the normal mucosa adjacent to adenoma or adenocarcinoma in overweight individuals indicates that obesity, especially abdominal obesity, increases the risk of tumorigenesis. Prostaglandins are important mediators of inflammation and tumorigenesis. Many types of research have shown that prostaglandin levels are different in various tumors, such as colorectal cancer, breast cancer, lung cancer, and ovarian cancer [47–49]. Notably, prostaglandins play an important role in the development of inflammation and colitis-associated cancer. Their receptor subtype prostaglandin E2 is mainly expressed in neutrophils and tumor-associated fibroblasts in the tumor microenvironment, has been found in colitis-associated colorectal tumor biopsies, and is positively correlated with cyclooxygenase-2 content [50]. The activation of prostaglandin E2 induced by a long-termed inflammation leads to Wnt/*β*-catenin signaling pathway activation, which plays an important role in the carcinogenesis of digestive system tumors [51]. Adenomatous polyposis coli (APC) is a component of the *β*-catenin destruction complex, which can accelerate the decomposition of *β*-catenin. The absence of APC inversely increases the incidence of CRC in Apc^Min/+^ mice.

The increase of cyclooxygenase-2 is positively correlated with tumorigenesis, and the inhibitory effect of NSAIDs on cyclooxygenases is well known. Long-termed administration of NSAIDs reduces the incidence of colitis, decreases the formation of aberrant crypt foci defined as the early stage of colon carcinoma, and lowers the adenomatous polyp burden in patients with familial adenomatous polyposis [52], but it increases the side effects of cardiovascular and digestive events, particularly gastrointestinal ulcers and bleeding [53]. The selective cyclooxygenase-2 inhibitor significantly reduced these side effects. It is generally believed that tumors originate from cancer stem cells. A recent study applying a selective cyclooxygenase-2 inhibitor to treat cancer stem cells eliminated early tumor regrowth [54]. Toll-like receptor-4 is associated with the activation of cyclooxygenase-2, the expression of downstream prostaglandins, and the stimulation of endogenous growth factor ligands and exerts important effects on colitis-associated CRC in ulcerative colitis [55]. The stimulation of toll-like receptor-4 by bacteria lipopolysaccharide or ROS activates the NF-*κ*B pathway. Hyperactivity of cyclooxygenase is partially mediated by the NF-*κ*B pathway. In addition, inflammatory cytokines such as interleukin-6 and tumor necrosis factor-*ɑ* synergistically launch the transcription of more diverse cytokines and cyclooxygenase and accelerate angiogenesis through the induction of hypoxia-inducible factor-1*ɑ*, thus accelerating the progress of inflammatory [56]. They form a positive feed-forward loop. However, free saturated fatty acids rather than polyunsaturated fatty acids (PUFAs) strengthened this process [57]. In contrast, a study by Larsson et al. [58] showed that *n*–3 PUFAs interfered with the activation of the NF-*κ*B pathway and competitively inhibited arachidonic acid metabolism, thus partially suppressing the inflammation.

### 2.5. Lipid Regulators

Dyslipidemia is highly associated with systematic metabolic diseases including cancer. Long-termed lipid challenges can aggravate chronic inflammation of gastrointestinal diseases. Nevertheless, the administration of anti-inflammatory drugs or lipid regulators can reduce the incidence of digestive tract cancer. The use of statins reduces morbidity and cancer-related mortality. This suggests that early intervention in the correction of lipids disorder is essential [59]. This also shows that early intervention is necessary to correct dyslipidemia. There is evidence that correcting dyslipidemia is beneficial to control inflammation and reduce the incidence of IBD and related tumors. A recent IBD cohort study showed that lipid-lowering drug statin reduced the incidence of colorectal tumors [60]. Statins competitively inhibit endogenous cholesterol synthesis rate-limiting enzyme HMG-CoA reductase, block intracellular mevalonate metabolism pathways, and lower intracellular cholesterol synthesis. More and more evidence shows that statins have many important antitumor effects. In addition, oxidized phospholipids damage cell membranes and significantly promote inflammatory response [61]. A study by Merwether et al. [62] demonstrated that apolipoprotein mimetics improved the inflammation of IBD induced by oxidized phosphatidylcholine. This may be explained by the combining of lipids with lipoproteins for transportation and subsequent metabolic shift. In addition, metabolites of short-chain fatty acid from fiber by digestive tract bacteria can maintain epithelial integrity by the receptor-activated manner and epigenetic modification [63]. Adjusting the diet structure by a complement of soluble fiber or omega-3 fatty acids improves inflammation response. Although they showed no benefits to patients with IBD [64], fiber-like pectin and cellulose indeed alleviated the severity of dextran sulfate sodium-induced colitis in mice [65]. Likewise, olive oil supplements improved the severity of dextran sulfate sodium-induced colitis in mice [66]. Furthermore, evidence showed that complement of soy isoflavones improved intestinal permeability, lowered serum lipid, inhibited inflammatory response, and corrected intestinal microbiota imbalance of obese rats [67].

## 3. Abnormalities of Lipid Metabolism in Tumor Cells Accelerate Disease Progress

### 3.1. Exogenous Lipid Uptake of Cancer

**Figure 1:**
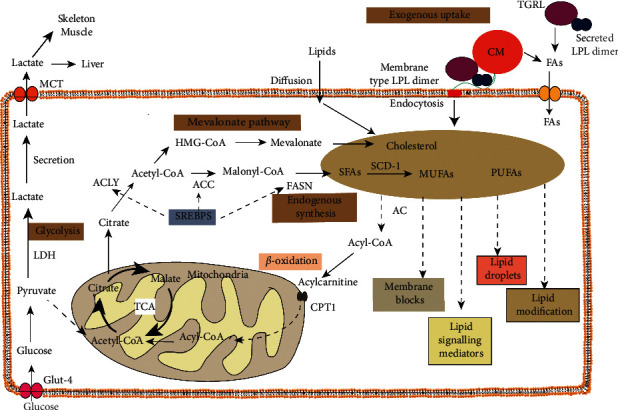
Lipid metabolism of cancer. Reprogrammed cancer cells not only acquire lipid species by uptake of exogenous lipids through diffusion and transmembrane channel CD36 but also endogenously synthesize fatty acids. The precursor mass of de novo synthesized fatty acids is initiated by pyruvate of glycolysis metabolites and NADPH of the pentose phosphate pathway. Following several key enzymes involved in lipogenesis, acetyl-CoA is aggregated as 16-carbon saturated palmitate. By the desaturation of SCD-1, the saturated fatty acid is converted into monounsaturated fatty acid, but polyunsaturated lipids are mostly acquired by exogenous supplements. These lipids function as lipid mediators in cellular signaling transduction, build cellular membrane blocks, modify substrates by lipid modification, and are directly utilized to generate ATP molecules by *β*-oxidation. Excess lipids are also stored in lipid droplets. FASN: fatty acid synthase; ACC: acetyl-CoA carboxylase; ACLY: ATP-citrate lyase; HMG-CoA: 3-hydroxy-3-methylglutaryl-CoA; SFAs: saturated fatty acids; MUFAs: monounsaturated fatty acids; PUFAs: polyunsaturated fatty acids; LDH: lactate dehydrogenase; TCA cycle: tricarboxylic acid cycle; SREBPS: sterol regulatory element-binding proteins; CM: chylomicrons; TGRL: triglyceride-rich lipoprotein lipase.

The lipid pools are mainly derived from endogenous synthesis and exogenous absorption. Quiescent cells usually consume and utilize exogenous fatty acids for degradation for energy. However, the direct absorption of exogenous fatty acids enables proliferating cells to bypass the activation of acetyl-CoA and modulate the source of lipid pool as an alternative manner to meet a high proliferation rate. Under certain nutritionally sufficient conditions, exogenous fatty acids are the first choice for membrane blocks over endogenous synthesis in certain proliferating cells, such as fibroblasts [68, 69]. Exogenous long-chain fatty acids are absorbed by the transmembrane channel cluster of differentiation 36 (CD36). Lipoprotein lipase (LPL), a dimerizing secretase, is a key enzyme for extracellular lipolysis, which catalyzes triglyceride-rich chylomicrons and very-low-density lipoprotein particles to become free fatty acids that are subsequently absorbed by CD36 in adipose and striated muscle [70]. LPL is highly expressed in adipose tissue, skeletal muscle, and the heart. It was reported that LPL exerts a noncatalytic function through receptor-mediated endocytosis to uptake and accumulate lipoproteins [71]. In addition, lipolysis, in some cancer cell lines, is another way for fatty acids acquisition. Some malignant tumors integrate the membrane surface with LPL rather than simply secreting it. The membrane-associated LPL dimers mediate extracellular hydrolysis (Figure 1: lipogenesis and Lipolysis), to convert the esterified fatty acid into free fatty acid, but also facilitate intracellular hydrolysis by acting as a nonenzymatic bridge. In addition, fatty acid transporters on the cellular membrane assist in the transport of extracellular fatty acids. At the same time, short-chain fatty acids and sterols can be uptaken in the form of free diffusion. Thus, they enable malignant tumor cells to acquire continuous fatty acids.

### 3.2. Endogenous Lipid Synthesis of Cancer

The most prominent and common characteristics of cancer are the novel metabolic reprogramming of aerobic glycolysis and lipid synthesis. Aerobic glycolysis, known as the “Warburg effect,” is a characteristic of most cancer cells and indeed all proliferating cells (such as activated immune cells). Tumors tend to synthesize endogenous lipids, using biomass from aerobic glycolysis of glucose, but also uptake extracellular fatty acids through membrane enzymes as an alternative source of lipid pools [72, 73]. This enables tumor cells to facilitate and incorporate glucose into biomass, making it fatty acids, amino acids, and nucleotides required for proliferation rather than efficient ATP generation [74]. Previous research by Reynier et al. [75] showed that, compared with colonic cells, undifferentiated human colon cancer cells exhibited elevated phospholipid profile with a significant difference of sphingomyelin and phosphatidylcholine, among which the composition of unsaturated fatty acid varied. The upregulated lipid metabolism required by the tumor is reflected by the overexpression and hyperactivity of various enzymes involved in lipogenesis and lipolysis (Figure 1: lipogenesis and lipolysis). Fatty acid synthase (FASN) is the rate-limiting enzyme for endogenous lipogenesis, which is essential for growth and proliferation. The increase of de novo synthetic lipids caused by the upregulation of FASN is one of the most noteworthy metabolic hallmarks of cancer [76]. Cancers such as colorectal carcinoma and prostate carcinoma show a transition to continuous fatty acid synthesis due to the significant increase and hyperactivity of lipid metabolic enzymes, regardless of the availability of extracellular lipids. However, except for the liver and adipose tissue with high expression of FASN, normal cells give priority to exogenous lipids [77]. Furthermore, monoacylglycerol lipase together with hormone-sensitive lipase catalyzes the degradation of triglyceride stored in intracellular lipid droplets, exerting a stream of intracellular free fatty acids. In the face of a harsh environment, tumor cells strengthen fat mobilization by releasing fatty acids from neutral lipids and obtaining large numbers of energy sources. Extracellular and intracellular fatty acids were esterified with the same neutral lipids or phospholipids and further incorporated into the membrane as cellular blocks in the correlation of proliferation rate. As lipid mediators, bioactive fatty acids also regulate signaling transduction through an array of signaling molecules [78]. Nevertheless, some fatty acids are directly transported and oxidized in mitochondria as energy sources. Meanwhile, excess fatty acids are stored in lipid droplets for restoring energy. In addition, increased lipid metabolism not only serves as a tool for membrane synthesis, but also affects cellular morphology and polarization. Hyperactivity of FASN can elicit Wnt-1 palmitoylation, resulting in continuous activation of *β*-catenin. In addition, ATP-citrate lyase (ACLY) catalyzes the formation of acetyl coenzyme A from citric acid, which is a precursor of mevalonate acid and fatty acid synthesis through successive condensation reactions and is overexpressed in colon cancer [79]. Acetyl coenzyme A is a substrate of acetylation modification as well. Acetylation modification of genes or proteins is important in cell biology. Posttranscriptional acetylation modification of histones mediates chromatin architectures and promotes gene expression.

Several studies have described special cellular mechanisms associated with lipids maintenance in tumor tissues [77, 79, 80]. These lipid-involved metabolic enzymes are affected by several factors, and their functions are reminiscent of the combination of a growth factor with its tyrosine kinases receptor to activate the PI3K/Akt pathway, which stimulates cell proliferation and survival. In this pathway, sterol regulatory element-binding protein-1 is a vital transcriptional factor associated with the gene transcription of intracellular lipid-relevant enzymes, under the regulation of the mammalian target of rapamycin complex-1 [81]. The activation of the mammalian target of rapamycin complex-1 stimulates the uptake of exogenous glucose, strengthens glycolysis, and increases the quantity of NADPH through an enhanced pentose phosphate pathway, which provides precursors for fatty acid synthesis. Although fatty acids are necessary for tumor progress, inhibitors of the PI3K/Akt pathway or chemical inhibitors and RNAi-mediated inhibition of key enzymes of lipid merely moderate tumor proliferation but do not play a significant role in tumor inhibition [82]. Interestingly, these effects can be partially restored by fatty acid supplementation, which implies a cross-talk network of tumors related to lipid metabolism control and emphasizes that the availability of exogenous fatty acids in the diet may disrupt the newly established balance of lipid homeostasis and exert detrimental effects of cancer therapy [83].

### 3.3. Unsaturated Lipid Metabolism of Cancer

The consumption of a diet high in red meat and low in fiber or fish may cause changes in the ratio of membrane polyunsaturated fatty acids to saturated fatty acids (P/S) [84]. The lower P/S ratio may contribute to the decreased fluidity of the membrane and the impaired function of the membrane. Phosphatidylcholine and phosphatidylethanolamine constitute the majority of membrane structures [85]. The fatty acid composition of these phospholipids significantly changes the fluidity of the membrane and subsequently influences cell metabolism. Overwhelmed palmitates, incorporated into phospholipid and triglyceride species of the membrane, increased the content of saturated lipid, which adversely affects the membrane movement and function of nonadipose tissue. It is well known that these accumulated saturated fatty acids and neutral lipids in nonadipose tissue rapidly stimulate apoptosis [86]. On the other hand, tumors detoxify their lipotoxicity by upregulating the activities of enzymes related to lipid metabolism. Stearoyl-CoA desaturase-1 (SCD-1) is the key enzyme that is responsible for the double bond formation of a stearoyl-CoA. Igal et al. proved that high expression and hyperactivity of SCD1 exist in the pathogenesis of many tumors [87], which may explain the high metabolic rate of tumors due to high membrane fluidity and nutrient substances communication. Both oleic and palmitoleic acid are important precursors for the biosynthesis of complex lipid species, including phospholipids, triglyceride, and cholesterol esters. In addition, lipid mediators usually contain a component of unsaturated fatty acid. The mammalian cell membrane is composed of a higher proportion of polyunsaturated fatty acids. It improves the fluidity of the cell membrane and causes the cell membrane to be more permeable, which is one of the key reasons for a high metabolic rate in mammals. What is more, SCD-1 accelerated the epithelial-mesenchymal transition by producing unsaturated fatty acids and inhibiting the activity of tumor suppressor PTEN [88]. Meanwhile, SCD-1 inhibitors reduced cell proliferation rate and caused a diminished capacity for anchorage-independent growth via the endoplasmic reticulum stress response mechanism for a higher proportion of saturated fatty acids of the endoplasmic reticulum membrane. The overlap of palmitate acid results in delayed cell replication due to endoplasmic reticulum stress [89]. In addition, evidence [79] showed that increased unsaturated fatty acids facilitated tumor growth and inhibited cellular apoptosis, which evoked tumor progress. We hypothesized that tumor cells with a high apoptotic threshold initiate cell death only when undergoing extreme stress. Thus, the imbalance of high proliferation and low apoptosis indicates tumor progress.

As shown above, the SCD-1 enzyme is essential for maintaining the homeostasis of fatty acid, and its imbalance will lead to lipid abnormality, thus interfering with the cellular physiological and biochemical activities. The deficiency of unsaturated fatty acids caused by SCD-1 inhibitors can induce apoptosis, especially in a hypoxic environment [90]. However, a study by Ducheix et al. [91] showed that oleic acids supplement inhibited inflammation and decreased crypt formation of SCD1^−/−^Apc^Min/+^ mice. To some extent, unsaturated fatty acids also exert antioxidative effects and reduce the incidence of stress-induced genomic damage. In addition, *n*−3 PUFA supplements may be beneficial to people with advanced cancer and cachexia and, to some degree, improve appetite, weight, and quality of life. The incorporation of *n*−3 PUFAs (such as docosahexaenoic acid) into the membrane can increase the activity of the insulin receptor and improve insulin sensitivity. It is suggested that PUFAs in the daily diet play a guiding role in the prevention of obesity and obesity-associated colon cancer [92].

## 4. Immunosuppression by Lipid Challenge Connives the Progress of Cancer

Lipid homeostasis plays an important role in immune function. The persistent lipid attack has an immunosuppressive effect on immune cells. The elevated absorption of exogenous lipid in dendritic cells can lead to the accumulation of lipid droplets, thereby impairing the antigen presentation of tumor immune to T lymphocyte [93]. It can be explained that the oxidized truncated lipids in the lipid droplets hurt the chaperone heat‐shock protein 70 through covalent binding, contributing to the dysfunction in the transport of peptide-MHC to the membrane surface [94]. Dysregulation of the immune system fails to execute immune surveillance and immune clearance against cancer, which provides opportunities for tumor development. A previous study by Luck et al. [95] proved that excessive fat intake altered the population of immune cells in the intestinal lamina propria and impaired immune functions of the intestine, which may lead to the occurrence of colorectal neoplasm. Similarly, Tie et al. [96] proved that high cholesterol levels eroded the hematopoietic stem cells through epigenetic modification, resulting in the decrease in the number and activity of the NKT and *γδ*T cells in the submucosa of the colon. Excessive fat intake not only promotes inflammation response but also accelerates colitis-related CRC by recruiting CC-chemokine-receptor-6 positive B cells and *γδ*T cells through ligand-receptor interaction [22].

Abnormal lipid metabolism has a negative impact on systemic immune monitoring and local immune cell function. Lipid metabolic disorder not only inhibits the systematic immune function but also suppresses the immune-infiltrating cell function in the tumor microenvironment by regulating the immunosuppressive function through the cross-talk between metabolic reprogramming tumor cells and surrounding stromal cells (such as M2 macrophages, monocytes), thereby accelerating the tumor progress. Tumor cells in the microenvironment deprive immune-infiltrating cells of energy supply by depleting glucose, thereby eroding the immunity [97], and trick them into the obesity trap by utilizing surrounding lipids as the energy source instead of glucose. Nevertheless, cholesterol is adequate in the microenvironment. The binding of cholesterol with the TCR receptors affected its multimerization and functional region, suppressed the immune activation of adaptive immunity, and enhanced the T-cell exhaustion. What is more, high cholesterol content restrained immune function by holding up the anticancer property of CD8^+^ T lymphocyte with a high expression of immune checkpoint programmed cell death protein-1 and T-cell immunoglobulin and mucin-domain containing-3 [98]. However, in response to available fat, tumor cells reprogramed their metabolism to better plunder energy-rich lipids and rapidly scavenged these lipids through continuous proliferation, which further deprived T lymphocyte of energy and accelerated the tumor progress. Furthermore, lipid droplets are a reservoir of inflammatory mediators and play an important role in tumor immunity in the interaction between tumors and stromal cells [99]. PGE2 derived of arachidonic acid in tumors promoted the resistance of immunotherapy [100]. In response to the effects of IFN-*γ*, the paracrine secreted tumor PGE2 promotes the translocation of p50 NF-*κ*B into the nuclear of monocytes and macrophages, thereby contributing to the production of immunosuppressive nitric oxide, which induced the immune dysfunction of T lymphocyte by interfering TNF-*α* [101]. Moreover, other than cytokine signaling in the microenvironment, unsaturated fatty acids themselves can also induce the phenotypic transition of myeloid cells to immunosuppressive M2-like macrophages [102].

**Figure 2:**
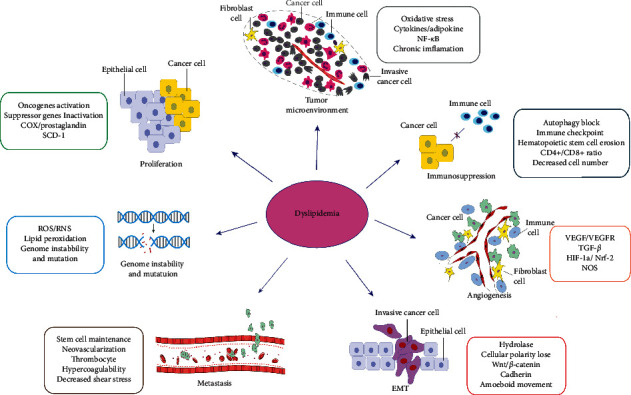
Schematic diagram of dyslipidemia upon colorectal cancer. This provides a comprehensive understanding of tumorigenesis and progress of cellular proliferation, angiogenesis, metastasis, migration, and immunosuppression. These insights will facilitate further developments of new drugs.

In addition, hyperlipidemia slows down the hemodynamics, accompanied by an increased platelet count in tumor patients, which leads to a hypercoagulability state. On the contrary, it provides the remaining tumor cells with a chance to survive and the distance metastasis of circulation cells. Furthermore, the chronic inflammatory of fatty liver disease increases the susceptibility of steatosis hepatocytes, affects the function of liver macrophages, and increases the risk of tumor liver metastasis. Similarly, a study by Ma et al. [103] showed that nonalcoholic fatty liver disease can affect the survival rate of CD4^+^ T lymphocytes and lead to a decrease in the CD4^+^/CD8^+^ ratio, which is mainly due to the high context of C18 : 2 fatty acids (especially linoleic acid). In addition, dietary changes affect the diversity of CD4^+^ T-cell receptors [104]. This may help reveal the tendency of liver metastasis of colorectal cancer (Figure 2: dyslipidemia upon colorectal cancer).

## 5. Conclusion and Perspective

In this paper, lipid components of unsaturated and saturated fatty acids, triglyceride, cholesterol, and phospholipid, as well as relevant enzymes to the occurrence and development of colitis-associated colorectal cancer, were exploited. Inflammatory bowel disease is more vulnerable to hyperlipidemia. Long-lasting intake of large amounts of fat can damage the epithelial junctions, alter the distribution of intestinal bacteria flora, and lead to lipopolysaccharide-induced endotoxemia and activation of the inflammatory pathway. It accelerates the injury of the mucosal epithelium and forms a vicious circle. The enhancement of oxidative stress and the turbulence of inflammatory mediators of the microenvironment have a great influence on the occurrence of tumors. In the repetitive phase of colitis, chronic inflammation enables these epithelial cells to continuously proliferate under the stimulation of prosurvival factors. The main purpose is to reconstruct the mucosa, but it is also implicated in the occurrence and malignancy of CRC.

The metabolic reprogramming of aerobic glycolysis and lipid synthesis converts raw carbohydrate and amino acid substrates into protein, nucleic acid, and lipid building blocks, which are required for sister cells. In the correlation of proliferation rate, esterified phospholipids are incorporated into the membrane as cell masses. Unsaturated fatty acids of phospholipids improve the fluidity of the cell membrane and cause permeable cell membranes, which is one of the key reasons for a high metabolic rate in mammals. In response to available fat, tumor cells reprogramed their metabolism to better plunder energy-rich lipids and rapidly scavenged these lipids through continuous proliferation. However, long-term lipid stimulation has an immunosuppressive effect on immune cells, resulting in immune surveillance and immune clearance dysfunction, which provides opportunities for tumor occurrence and distant metastasis. Lipid metabolic disorder not only inhibits the systematic immune function but also suppresses the immune-infiltrating cell function in the tumor microenvironment by regulating the immunosuppressive function through the cross-talk between metabolic reprogramming tumor cells and surrounding stromal cells, thereby accelerating the tumor progress.

In the absence of carcinogenic stimulation, a high-fat diet did not increase the incidence of obesity-related CRC in wild-type mice. However, for the precancerous lesion model of Apc^Min/+^ mice and azoxymethane-induced colitis, excess fat increased the incidence of colon tumors [105]. Likewise, long-lasting high-fat uptake accelerated the progress of the colitis-associated colorectal tumor. Visceral adipocytes may participate in establishing a favorable tumor microenvironment and play a role in the occurrence of colorectal neoplasm. Furthermore, obesity-associated insulin resistance aggravates both glucose and lipid metabolic disorders, which are key factors to tumor metabolism and eventually promote the progress of tumors.

Arachidonic pathway inhibitors of NSAIDs have been shown to efficiently decrease the formation of aberrant crypt foci, lower the burden of the adenomatous polyp, and reduce the incidence of CRC. Nevertheless, lipid regulators can reduce the incidence of colitis-associated CRC. This indicates that the tumorigenesis of colitis-associated CRC is closely related to intestinal inflammation and lipid abnormality. It is of practical significance to reduce the formation of intestinal tumors and lower the burden of tumors by intervening with the inflammatory response and the disorder of lipid metabolism.

The lipogenesis-relevant enzymes in CRC elicited by the reprogramed metabolism are required to acquire energy to meet the high proliferation rate of cells and to help cells survive even in a harsh environment. Tumors mainly facilitate the synthesis of endogenous fatty acids by FASN and absorb extracellular fatty acids as another way of lipid pools. High expression and hyperactivity of SCD-1 may be one of the reasons to explain the high metabolic rate of tumors because of its high membrane fluidity and nutrient substances communication. Tumor lipid metabolic reprogramming, in turn, affects serum cholesterol and high-density lipoprotein level [106]. However, we speculate that the enzymes involved in lipid metabolism can be potential therapeutic targets for cancer. Chemical inhibition or RNAi-mediated suppression of key enzymes in lipid metabolism can slow down tumor proliferation and promote apoptosis but can be rescued by the supplement of fatty acids. Therefore, diet restriction and moderate physical activity are essential. Despite therapeutic methods on these targeted key enzymes to retain the accumulation of lipid mass, targeting potential lipid scavenging ways may work simultaneously. In addition, the application of colon endoscopic screening is warranted to detect precancerous and malignant lesions to reduce the morbidity and mortality of CRC, marking the cornerstones of cancer prevention. This paper was concentrated on colitis-associated CRC, focused on the cancer-promoting effect of abnormal lipid metabolism on colitis, and analyzed the response of colorectal cancer cells and the tug-of-war between tumor cells and immune cells to the high fat, thus providing a comprehensive understanding of the influence of dyslipidemia on tumorigenesis and a prospect for the development of lipid disorder on the tumor immunity. These opinions summarize the overall understanding of dyslipidemia in tumor biology and are expected to promote further development of new anticancer drugs.

Tumor exploits several novel mechanisms to cope with therapeutic pressure and promote deterioration, adding the challenge of antitumor. There is evidence that excessive saturated lipids packed more densely and altered the kinetics of lateral and transverse membrane due to the upregulation of FANS or the RNAi-mediated SCD-1, resulting in reduced membrane permeability and drug resistance, highlighting the challenges of anticancer drug therapy [107]. In addition, tumor cells take full advantage of autologous substances to survive even in the hard environment, known as autophagy, by systematically swallowing and decomposing cellular components, such as lipid droplets and misfolded proteins, to support a dynamic recycling system to maintain ATP, which is generally maintained at basal levels. Furthermore, the liver is the most common target viscus for distant metastasis of digestive tract cancer on account of special anatomy. It is significantly urgent to uncover the mechanism by which nonalcoholic fatty liver disease and resultant liver cirrhosis suffer higher risks of CRC liver metastasis as well. There is still more that we should focus on, and more effective measures should be taken.

## Data Availability

All data used in this study are included within the article.
